# ROCK1 Mediates Retinal Glial Cell Migration Promoted by Acrolein

**DOI:** 10.3389/fmed.2021.717602

**Published:** 2021-09-03

**Authors:** Kanae Fukutsu, Miyuki Murata, Kasumi Kikuchi, Shiho Yoshida, Kousuke Noda, Susumu Ishida

**Affiliations:** Laboratory of Ocular Cell Biology and Visual Science, Department of Ophthalmology, Faculty of Medicine and Graduate School of Medicine, Hokkaido University, Sapporo, Japan

**Keywords:** rho-associated coiled-coil-containing protein kinase 1, acrolein, retinal glial cells, cell migration, diabetic retinopathy

## Abstract

**Objective:** Acrolein is a highly reactive aldehyde that covalently binds to cellular macromolecules and subsequently modulates cellular function. Our previous study demonstrated that acrolein induces glial cell migration, a pathological hallmark of diabetic retinopathy; however, the detailed cellular mechanism remains unclear. The purpose of this study was to investigate the role of acrolein in retinal glial cell migration by focusing on rho-associated coiled-coil-containing protein kinases (ROCKs).

**Methods:** Immunofluorescence staining for ROCK isoforms was performed using sections of fibrovascular tissue obtained from the eyes of patients with proliferative diabetic retinopathy (PDR). Rat retinal Müller glial cell line, TR-MUL5, was stimulated with acrolein and the levels of ROCK1 were evaluated using real-time PCR and western blotting. Phosphorylation of the myosin-binding subunit of myosin light chain phosphatase [myosin phosphatase target subunit 1, (MYPT1)] and myosin light chain 2 (MLC2) was assessed. The cell migration rate of TR-MUL5 cells exposed to acrolein and/or ripasudil, a non-selective ROCK inhibitor, was measured using the Oris cell migration assay.

**Results:** ROCK isoforms, ROCK1 and ROCK2, were positively stained in the cytosol of glial cells in fibrovascular tissues. In TR-MUL5 cells, the mRNA expression level of *Rock1*, but not *Rock2*, was increased following acrolein stimulation. In line with the PCR data, western blotting showed increase in ROCK1 and cleaved ROCK1 protein in TR-MUL5 cells stimulated with acrolein. N-acetylcysteine (NAC) suppressed acrolein-associated *Rock1* upregulation in TR-MUL5 cells. Acrolein augmented the phosphorylation of MYPT1 and MLC2 and increased the cell migration rate of TR-MUL5 cells, both of which were abrogated by ripasudil.

**Conclusions:** Our study demonstrated that ROCK1 mediates the migration of retinal glial cells promoted by the unsaturated aldehyde acrolein.

## Introduction

Diabetic retinopathy (DR) is one of the leading causes of blindness worldwide (1). During its advanced stage, pathological neovascularization due to retinal ischemia causes the formation of fibrovascular tissues at the vitreoretinal surface, which is a hallmark of proliferative diabetic retinopathy (PDR). Vitreous traction against fibrovascular tissues subsequently leads to severe complications such as vitreous hemorrhage and tractional retinal detachment in PDR, both of which, without adequate treatment, eventually result in severe visual impairment. Therefore, the mechanisms implicated in the development of fibrovascular tissue are of great significance to both clinicians and researchers.

Fibrovascular tissue consists of small new vessels, extracellular matrix, and cellular components, such as inflammatory cells, fibroblasts, and glial cells (2). In the eye, there are two basic types of macroglial cells: Müller glial cells and astrocytes (3). Of these, Müller glial cells, which provide homeostatic, metabolic, and functional support to neurons, can become activated upon pathogenic stimuli (3) and migrate toward the vitreoretinal surface in the diabetic retina (4). Since activated Müller glial cells produce a variety of inflammatory cytokines including vascular endothelial growth factor (VEGF) (5, 6), it has been presumed that migrated Müller glial cells are one of the major participants in the formation of fibrovascular tissue. We previously investigated the triggers that facilitate the migratory response of Müller glial cells and found that acrolein, an unsaturated aldehyde, was one of the causative factors (7). Acrolein is a highly reactive aldehyde distributed in air pollutants such as cigarette smoke (8) and is associated with the increase in oxidative stress by reducing the antioxidant glutathione (GSH) in retinal capillary endothelial cells (9). In Müller glial cells, acrolein is generated through polyamine metabolism under hypoxic conditions (10) and accelerates cellular motility by inducing chemokine (CXC motif) ligand 1 (CXCL1) in an autocrine fashion (7). Therefore, a growing body of evidence explicitly suggests that acrolein is one of the trigger stimuli for glial cell migration in the diabetic retina and therefore, plays a role in the cell motility mechanism.

Rho-associated coiled-coil-containing protein kinases (ROCKs) are ubiquitously expressed serine-threonine kinases, which represent the main effector proteins of the RhoA and RhoC pathways (11). In humans, ROCKs exist in two isoforms, i.e., ROCK1 and ROCK2, and these enzymes mainly regulate the organization of the actin cytoskeleton and associated dynamic events such as cell contraction and migration (12). Among more than 30 common ROCK substrates, one of the most described targets is the myosin phosphatase target subunit 1 (MYPT1) of the myosin light chain phosphatase (MLCP) (12). ROCK-mediated phosphorylation of MYPT1 hampers the catalytic activity of MLCP, in turn resulting in an increase in myosin light chain (MLC) phosphorylation and subsequent cell contraction. Previously, it was reported that cigarette smoking induces barrier dysfunction through the ROCK pathway in lung microvascular cells, indicating that the acrolein found in cigarette smoke acts upstream of the ROCK cascade. In addition, it was demonstrated that ROCK is involved in leukocyte-induced diabetic retinal endothelial injury (13) and inflammatory microvascular damage (14). Lines of evidence indicate that ROCK participates particularly in the vascular injury and plays a role in the pathogenesis of PDR. By contrast, the role of ROCKs and its associated pathways in retinal glial cells, which also participate in the progression of DR, remains uninvestigated.

In this study, we explored the regulatory mechanisms responsible for Müller glial cell migration induced by the unsaturated aldehyde acrolein, especially focusing on ROCKs.

## Materials and Methods

### Specimens and Materials

Fibrovascular tissues surgically removed from patients with PDR were used for immunofluorescence microscopy. All experiments were conducted in accordance with the tenets of the Declaration of Helsinki, following approval from the Institutional Review Committee of Hokkaido University Hospital (IRB #014-0293). Written informed consent was obtained from all the patients after explaining the purpose and procedures of this study. Ripasudil, a ROCK inhibitor, was provided by Kowa Company, Ltd. (Tokyo, Japan).

### Cell Culture

Conditionally immortalized rat retinal Müller cell line TR-MUL5 from transgenic rats harboring the temperature-sensitive SV 40 large T-antigen gene was provided by Fact Inc. (Sendai, Japan) (15). TR-MUL5 cells were cultured at 33°C in Dulbecco's modified Eagle's medium (Fuji Film Wako Pure Chemicals, Osaka, Japan), supplemented with 10% fetal bovine serum (Thermo Fisher Scientific, Waltham, MA, USA) in an atmosphere of 95% air and 5% CO_2_.

### Immunofluorescence Microscopy

Paraffin sections of fibrovascular tissues were deparaffinized and hydrated *via* exposure to xylene and graded alcohols, followed by water. After microwave-based antigen retrieval in 10 mM citrate buffer (pH 6.0), the paraffin sections were incubated in 10% normal goat serum (Thermo Fisher Scientific) for 30 min and then incubated overnight with a primary rabbit monoclonal antibody against ROCK1 (1:50, EP786Y, Abcam, Cambridge, MA, USA) and mouse monoclonal antibody against GFAP (1:50, NCL-L-GFAP-GA5, Leica Biosystems, Wetzlar, Germany), or mouse monoclonal antibody against ROCK2 (1:100, 610623, BD, Franklin Lakes, NJ, USA) and rabbit polyclonal antibody against GFAP (1:200, Z033429, Agilent Technologies, Inc., Santa Clara, CA, USA) at 4°C, prior to exposure to Alexa Fluor 546 goat anti-rabbit IgG and Alexa Fluor 488 goat anti-mouse IgG, or Alexa Fluor 488 goat anti-rabbit IgG and Alexa Fluor 546 goat anti-mouse IgG (1:500, Thermo Fisher Scientific) for 1 h at room temperature. Serial sections were incubated with normal rabbit IgG (Abcam) and normal mouse IgG (Agilent) as negative controls.

For immunocytochemistry, TR-MUL5 cells were seeded into a 6-well plate with a cover glass and incubated for 24 h. The cells were serum-starved for 17 h and stimulated with acrolein for 23 h, followed by addition of ripasudil (2 μM) and incubation for 1 h. The cells were fixed with 4% paraformaldehyde for 15 min and permeabilized with 0.1% Triton X-100 for 10 min. Cells were incubated in 10% normal goat serum for 30 min and then incubated with a primary mouse monoclonal antibody against phosphorylated MLC2 (p-MLC2, 1:100, #3675, Cell Signaling, Danvers, MA, USA) at 4°C overnight, prior to exposure to Alexa Fluor 546 goat anti-mouse IgG (1:400) and phalloidin (1:143, Cytoskeleton, Inc. Denver, CO, USA) for 1 h at room temperature.

Nuclei were counterstained with 4′,6-diamidino-2-phenylindole (DAPI; Roche Applied Science, Indianapolis, IN, USA), and photomicrographs were taken with a fluorescence microscope (BIOREVO BZ-9000, Keyence, Osaka, Japan).

### Quantitative Real-Time PCR

The expression levels of *Rock1, Rock2, Mypt1*, and *Mlc2* mRNA were examined with quantitative real-time PCR. TR-MUL5 cells were seeded into a 6-well plate at a density of 4 × 10^5^ cells per well and incubated for 24 h. The cells were serum-starved for 17 h and stimulated with acrolein for 6 h with or without N-acetylcysteine (NAC) pre-treatment for 30 min. Another set of the cells were also serum-starved for 17 h and stimulated with H_2_O_2_ (100 μM) for 6 h as a positive control. Total RNA was extracted from cells using TRI reagent (Molecular Research Center, Inc., Cincinnati, OH, USA) and reverse transcribed to cDNA using GoScript reverse transcriptase (Promega, Madison, WI, USA), according to the manufacturer's protocol. Analysis of mRNA levels was performed on a StepOnePlus Real-Time PCR system (Thermo Fisher Scientific) using GoTaq qPCR Master Mix (Promega). The primer sequences used for real-time PCR and the expected size of the amplified products were as follows: 5′-ATGAACTTCAAATGCAGTTGGCT-3′ (forward) and 5′-AATAAGGAATGCAGGCAGAACCA-3′ (reverse) for rat *Rock1* (NM_001389239.1), 156 bp; 5′-CTGCTGACTGAGCGAACACT-3′ (forward) and 5′- ACCACGCTTGACAGGTTCTT-3′ (reverse) for rat *Rock2* (NM_013022.2), 84 bp; 5′-GCCTTGCCCTCAGAGGATCTA-3′ (forward) and 5′-CATTGGAGCTCCCTTCTGCTG-3′ (reverse) for rat *Mlc2* (*Myl2*, NM_001035252.2), 77 bp; 5′-GTCAGCTCAACAGGCCAAAC-3′ (forward) and 5′-TCGCCGTCGTTCTCTGATTG-3′ (reverse) for rat *Mypt1* (*Ppp1r12a*, NM_053890.2), 152 bp; 5′-GGGAAATCGTGCGTGACATT-3′ (forward) and 5′-GCGGCAGTGGCCATCTC-3′ (reverse) for rat *Actb* (NM_031144), 76 bp. The PCR conditions used were 95°C for 2 min, followed by 95°C for 15 s and 40 cycles of 60°C for 1 min. All data were calculated using the ΔΔCt method, with the level of *Actb* mRNA as a normalization control.

### Western Blotting

TR-MUL5 cells were seeded into a 6-cm dish at a density of 8 × 10^5^ cells per dish and incubated for 24 h. The cells were serum-starved for 17 h and stimulated with acrolein for 24 h. For MYPT1 and MLC2 western blotting, ripasudil (0.08, 0.4, or 2 μM) was added 1 h before cell collection. TR-MUL5 cells were lysed in 1x SDS sample buffer [62.5 mM Tris-HCl (pH 6.8), 2%SDS, 10% glycerol, phosphatase inhibitor cocktail (PhosSTOP, Merck, Burlington, MA, USA) and protease inhibitor cocktail (Complete mini, Merck)]. The cell lysates were sonicated thrice for 5 s each on ice and centrifuged at 15,000 × *g* at 4°C for 10 min. Protein concentration was measured using a BCA protein assay kit (Thermo Fisher Scientific) and adjusted to 2 mg/mL with 0.01% bromophenol blue and 5% 2-mercaptoethanol. The samples were boiled at 95°C for 3 min, separated using SDS-PAGE, and electroblotted onto polyvinylidene fluoride membranes (Merck). Membranes were incubated with 5% skim milk for 1 h and then incubated with primary antibodies against ROCK1 (1:5000, EP786Y, Abcam), MLC2 (1:1000, #8505, Cell Signaling), p-MLC2 (1:1000, #3675, Cell Signaling), MYPT1 (1:1000, #2634, Cell Signaling), pMYPT1 (1:1000, #5163, Cell Signaling), and cleaved ROCK1 (1:1000, 154C1465, Novus biologicals, Centennial, CO, USA) at 4°C overnight, followed by incubation with goat anti-mouse IgG (H+L) horseradish peroxidase conjugate (1:4000, Jackson ImmunoResearch Laboratories, Inc., West Grove, PA, USA) or goat anti-rabbit IgG (H+L) horseradish peroxidase conjugate (1:4000, Jackson Immunoresearch) at room temperature for 1 h. Signals were visualized using a SuperSignal West Pico Chemiluminescent Substrate (Thermo Fisher Scientific).

### GSH Assay

TR-MUL5 cells were seeded into a 24-well plate at a density of 1 × 10^5^ cells per well and incubated for 24 h. The cells were serum-starved for 17 h and stimulated with 10–50 μM acrolein for 3 h. Total GSH levels were measured using a total GSH assay kit (Nikken Seil Co., Ltd., Shizuoka, Japan), according to the manufacturer's protocol.

### Migration Assay

Migration capacity was assessed using the Oris™ Cell Migration Assay kit (Platypus Technologies, Madison, WI, USA) according to the manufacturer's protocol. Briefly, TR-MUL5 cells were seeded into a collagen I-coated 96-well Oris™ plate at a density of 1 × 10^5^ cells per well and incubated for 24 h at 33°C in an atmosphere of 95% air and 5% CO_2_. The cells were starved for 17 h and stimulated with 25 μM acrolein for 24 h. Ripasudil (0.08, 0.4, or 2 μM) and the DNA synthesis inhibitor aphidicolin (10 μg/mL) were added to the cells 1 h prior to the start of migration. Silicone stoppers were then removed to allow cell migration to the central detection zone for 24 h at 33°C in an atmosphere of 95% air and 5% CO_2_. The cell migration area was evaluated using BIOREVO BZ-9000 (Keyence) and analyzed with a BZ-II analyzer (Keyence).

### Statistical Analysis

All results are presented as the mean ± SEM. Student's *t*-test was used for pairwise statistical comparisons between groups, and one-way ANOVA, followed by the *post-hoc* Tukey-Kramer test, if appropriate, was used for multiple comparisons. Differences in the means were considered statistically significant at *P* < 0.05.

## Results

### Tissue Localization of ROCKs in Fibrovascular Tissues of PDR

**Figure 1:**
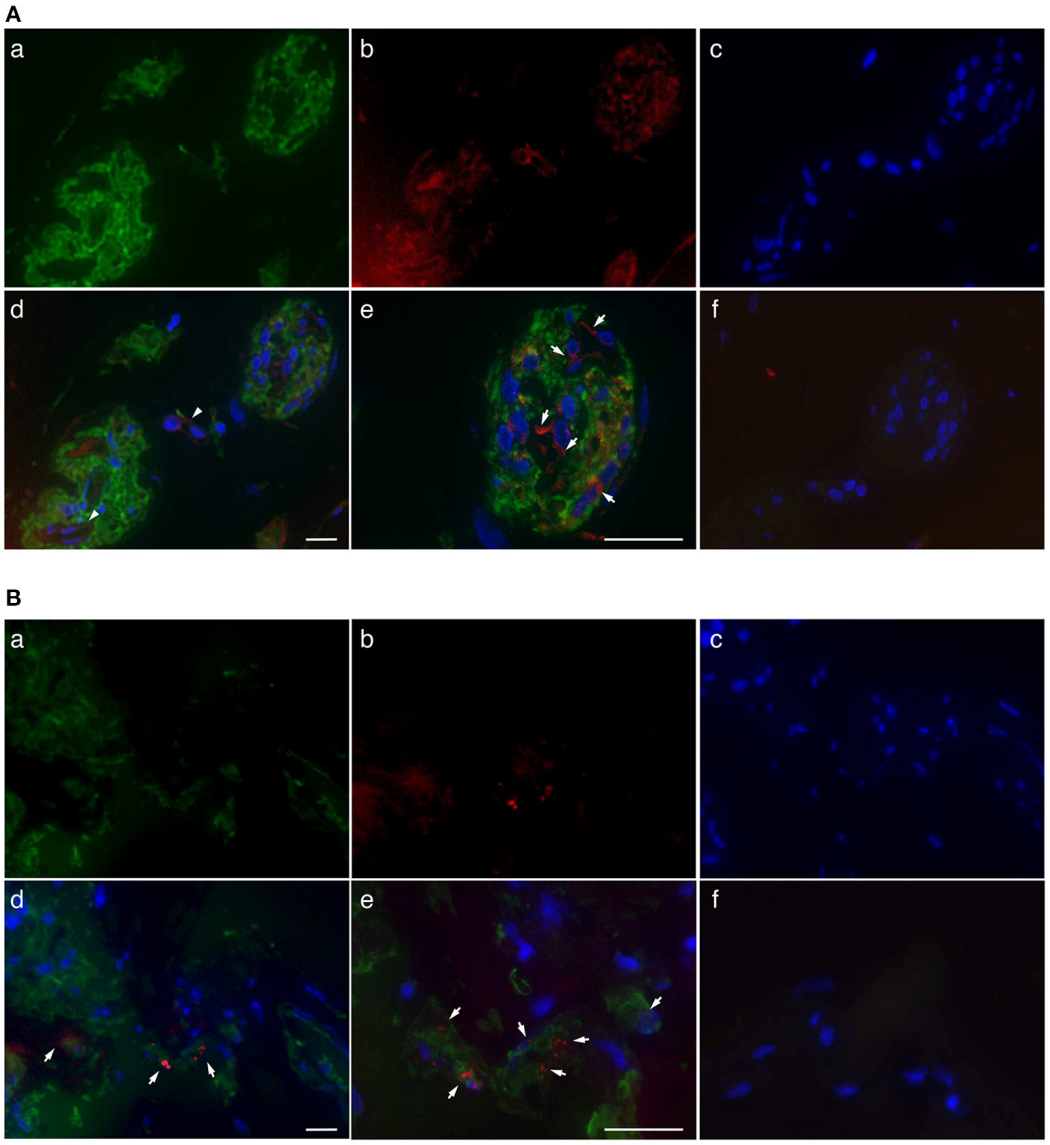
Localization of ROCKs in GFAP-positive cells in fibrovascular tissues of patients with PDR. **(A)** Representative fluorescent micrographs of ROCK1 immunofluorescence in fibrovascular tissues. (a) Green, GFAP (Alexa Fluor® 488). (b) Red, ROCK1 (Alexa Fluor® 546). (c) Blue, nuclei counterstained with DAPI. (d) Merged image. Arrows indicate the co-localization of ROCK1 with GFAP in the fibrovascular tissue. (e) High-magnification image. (f) Negative control (mouse and rabbit IgG) in sequential sections. **(B)** Representative fluorescence micrographs of ROCK2 immunofluorescence in fibrovascular tissues. (a) Green, GFAP (Alexa Fluor® 488). (b) Red, ROCK2 (Alexa Fluor® 546). (c) Blue, nuclei counterstained with DAPI. (d) Merged image. Arrows indicate co-localization of ROCK-2 with GFAP in fibrovascular tissue. (e) High-magnification image. (f) Negative control (mouse and rabbit IgG) in sequential sections. Scale bars, 25 μm.

Immunofluorescence staining was performed to determine the localization of ROCKs in fibrovascular tissues obtained from patients with PDR. ROCK1 staining was observed in clusters of GFAP-positive cells and vascular structures in fibrovascular tissues (Figure 1A). Similarly, ROCK2 signals were found in clusters of GFAP-positive cells; however, its fluorescence staining intensity was relatively faint (Figure 1B). High-magnification images revealed that immunoreactivity of ROCK1 and ROCK2 was the most abundant in the areas surrounding the nuclei (Figures 1A,B). These data indicate that ROCKs are predominantly localized in the cytoplasm of glial cells in fibrovascular tissues.

### Acrolein Induces ROCK1 Production in Retinal Glial Cells

**Figure 2:**
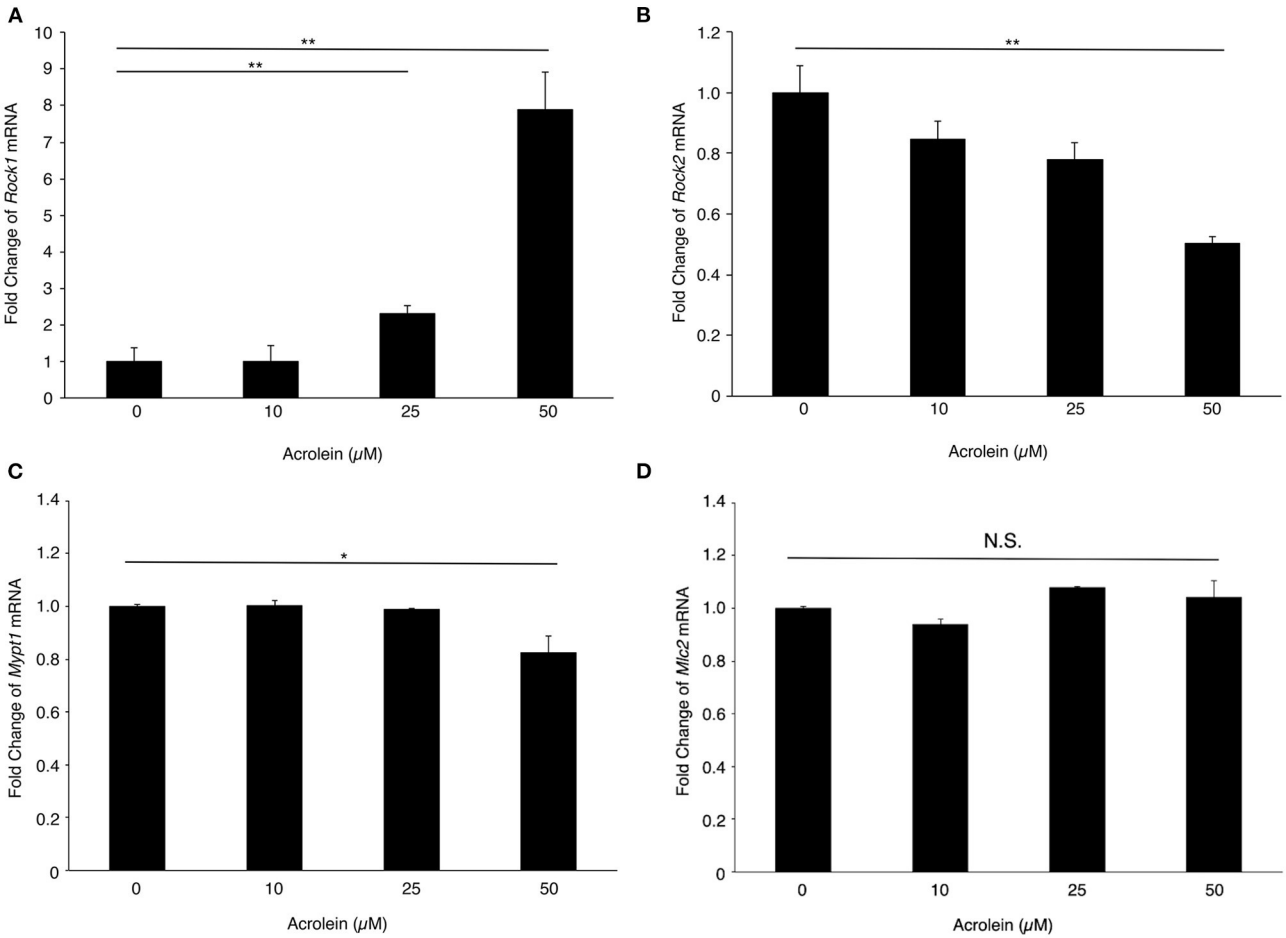
Effect of acrolein on transcriptional levels of *Rocks, Mypt1*, and *Mlc2* in TR-MUL5 cells. The mRNA expression levels of **(A)** *Rock1*, **(B)** *Rock2*, **(C)** *Mypt1*, and **(D)** *Mlc2* when TR-MUL5 cells were incubated with acrolein (0–50 μM, *n* = 6 each). Values represent mean ± SEM; *P < 0.05; **P < 0.01; N.S., not significant.

To evaluate the changes in expression of ROCKs and its associated molecules in Müller glial cells stimulated with acrolein, the mRNA levels of *Rock1, Rock2, Mypt1*, and *Mlc2* mRNA were assessed. *Rock1* mRNA expression was significantly increased by acrolein stimulation in a dose-dependent manner (*n* = 3 each, *P* < 0.01, Figure 2A), whereas *Rock2* mRNA expression decreased in response to acrolein exposure (Figure 2B). In addition, the mRNA levels of *Mypt1* and *Mlc2* were not changed after acrolein stimulation (Figures 2C,D).

**Figure 3:**
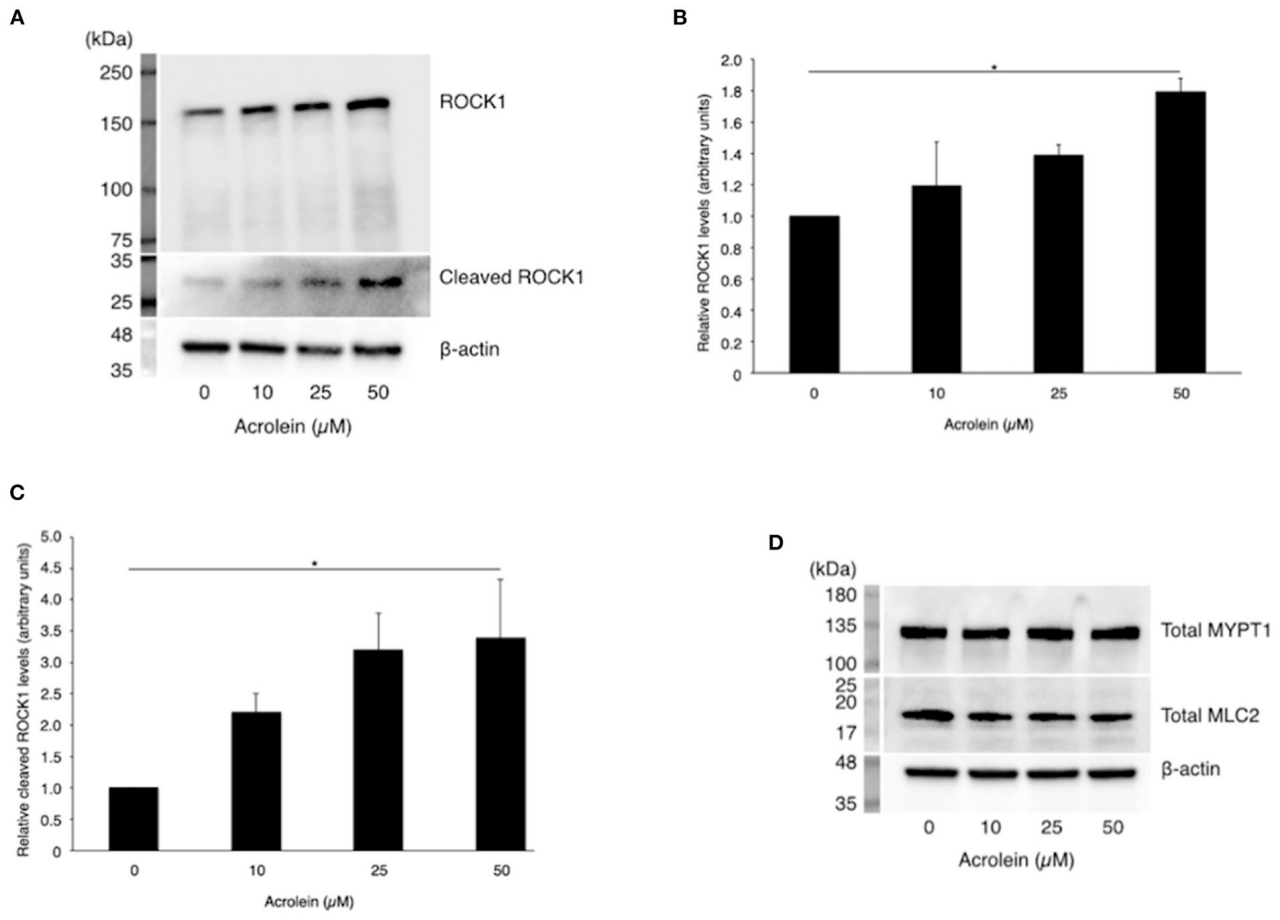
Effect of acrolein on protein levels of ROCK1, MYPT1, and MLC2 in TR-MUL5 cells. **(A)** Western blotting of total and cleaved forms of ROCK1 protein when TR-MUL5 cells were incubated with acrolein (0–50 μM). **(B,C)** Densitometric analysis of the bands. **(B)** Total forms of ROCK1 protein (0–50 μM, *n* = 3 each). **(C)** Relative cleaved forms of ROCK1 protein (0–50 μM, *n* = 4 each). **(D)** Total protein of MYPT1 and MLC2 in TR-MUL5 cells exposed to acrolein (0–50 μM). Values represent mean ± SEM; *P < 0.05.

In accordance with real-time PCR data, western blotting (Figure 3A) revealed an increase in ROCK1 production in TR-MUL5 cells stimulated with acrolein (*n* = 3 each, *P* < 0.05, Figure 3B). Furthermore, acrolein increased cleaved form of ROCK1 in a dose-dependent manner (*n* = 4 each, *P* < 0.05, Figures 3A,C). The protein levels of MYPT1 and MLC2 showed no increase with acrolein stimulation (Figure 3D).

### Oxidative Stress Mediates ROCK1 Production in Retinal Müller Glial Cells Under Acrolein Stimulation

**Figure 4:**
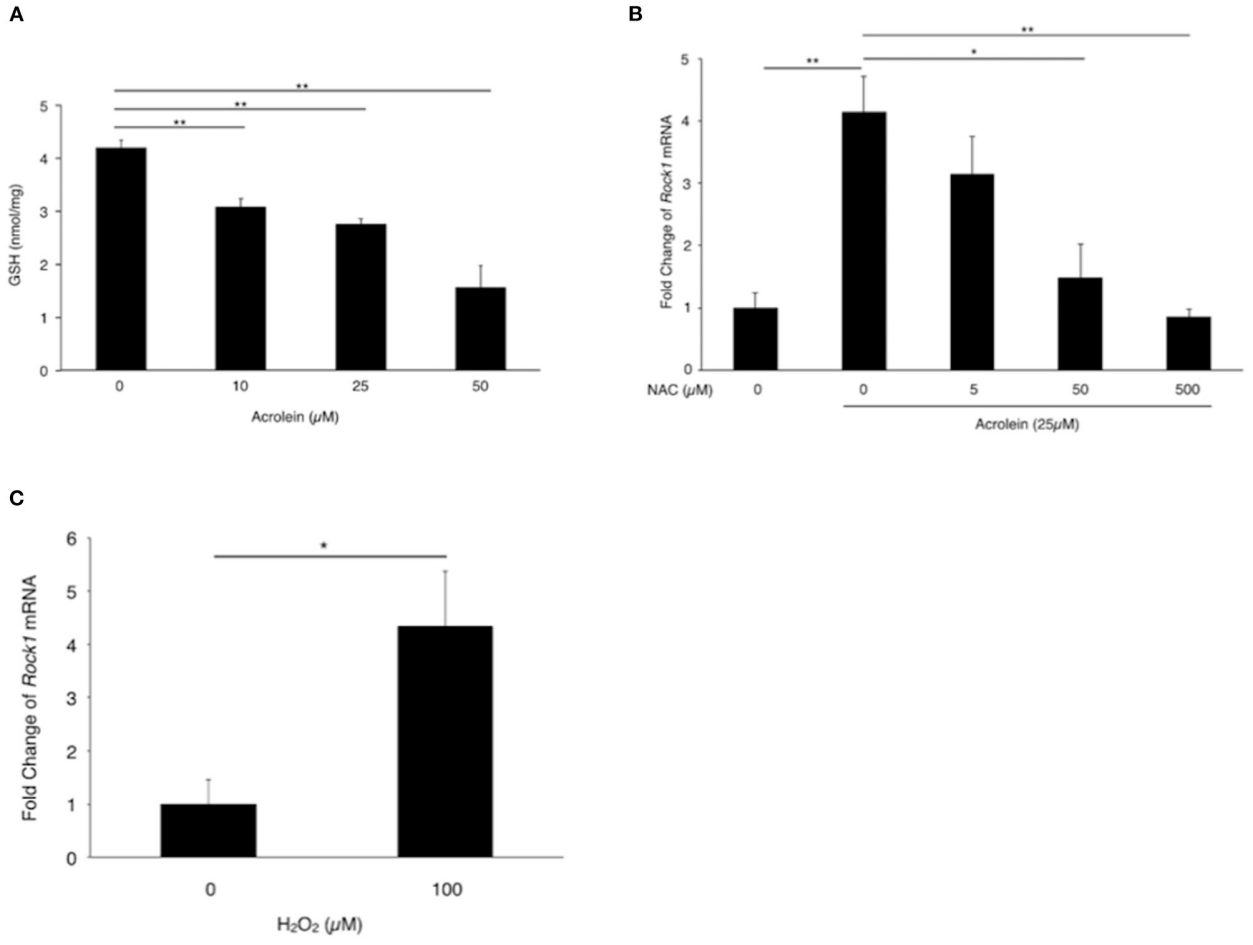
Impact of acrolein stimulation on antioxidant defense systems in TR-MUL5 cells. **(A)** Total GSH levels when TR-MUL5 cells were exposed to acrolein for 3 h. **(B)** *Rock1* mRNA levels in acrolein-stimulated TR-MUL5 cells with or without treatment of N-acetylcysteine (NAC) (*n* = 3 each). **(C)** *Rock1* mRNA levels in TR-MUL5 cells treated with or without hydrogen peroxide (*n* = 3 each). Values represent mean ± SEM; *P < 0.05; **P < 0.01.

Based on the real-time PCR results, we focused on ROCK1 and examined whether oxidative stress is involved in acrolein-induced ROCK1 production in Müller glial cells. Total GSH levels were reduced from 4.19 nmol/mg to 1.56 nmol/mg in TR-MUL5 cells after acrolein stimulation (*n* = 4, *P* < 0.05, Figure 4A). Furthermore, ROCK1 expression showed an ~4-fold increase in TR-MUL5 cells stimulated with acrolein compared to the control cells, while the increase was abolished by NAC in a dose-dependent manner (*n* = 3, *P* < 0.01, Figure 4B). In response to hydrogen peroxide, ROCK1 expression showed an ~4-fold increase in TR-MUL5 cells compared with the control (*n* = 3, *P* < 0.05, Figure 4C).

### ROCK1-pMYPT1-pMLC Pathway Is Activated by Acrolein

**Figure 5:**
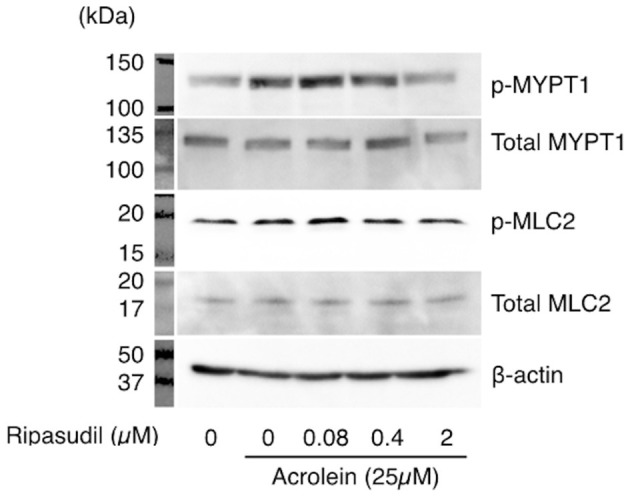
Changes in phosphorylation of MYPT1 and MLC2 in TR-MUL5 cells treated with acrolein and ripasudil. Phosphorylation levels of MYPT1 and MLC2 in TR-MUL5 cells after acrolein stimulation. Ripasudil suppressed the increase of phosphorylated forms of MYPT1 and MLC2 induced by acrolein stimulation in TR-MUL5 cells.

To examine the impact of acrolein and ripasudil on ROCK activity, phosphorylation of MYPT1 and MLC2 was assessed in TR-MUL5 cells. As shown in Figure 5, phosphorylation of both MYPT1 and MLC2 showed increase in TR-MUL5 cells when stimulated with acrolein, while the total protein levels of MYPT1 and MLC2 were unchanged (Figure 3D). In addition, ripasudil reduced the acrolein-induced phosphorylation of MYPT1 and MLC2 (Figure 5).

**Figure 6:**
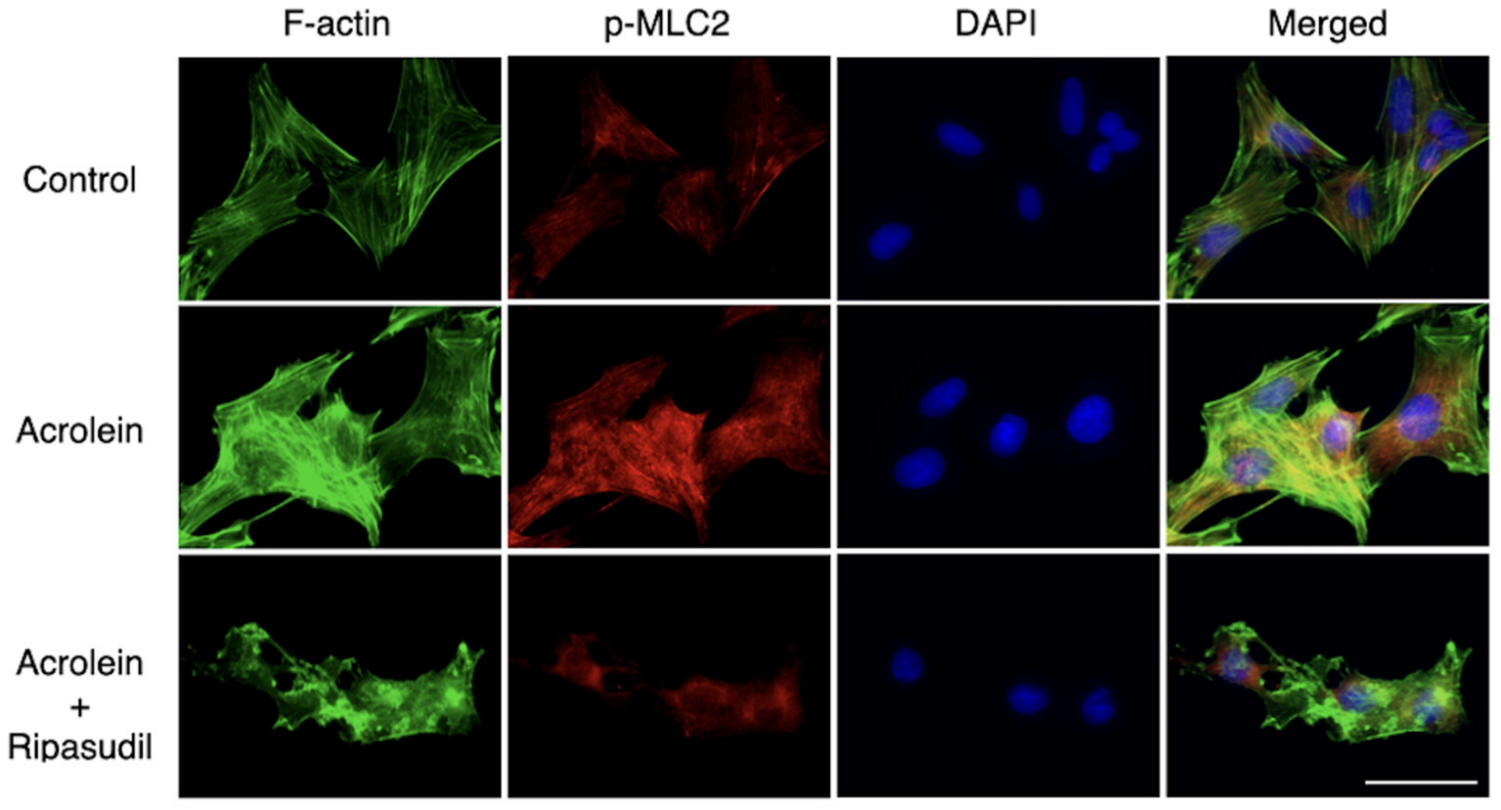
Changes in cellular morphology and stress fiber formation in TR-MUL5 cells treated with acrolein and ripasudil. Representative fluorescent staining patterns of F-actin (green, Acti-stain^TM^ 488) and p-MLC2 (red, Alexa Fluor® 546) in TR-MUL5 cells treated with acrolein (25 μM) and/or ripasudil (2 μM). Blue, nuclei counterstained with DAPI. Scale bar, 50 μm.

As shown in Figure 6, immunofluorescence microscopy revealed that acrolein stimulation increased organization of actin stress fibers in TR-MUL5 cells, whereas actin depolymerization was observed in the cells treated with ripasudil. The staining signal of p-MLC2 increased with acrolein stimulation along with actin fibers, and it was attenuated in the presence of ripasudil. Additionally, ripasudil administration caused cell body shrinkage in TR-MUL5 cells.

### Ripasudil Attenuates Glial Cell Migration Induced by Acrolein

**Figure 7:**
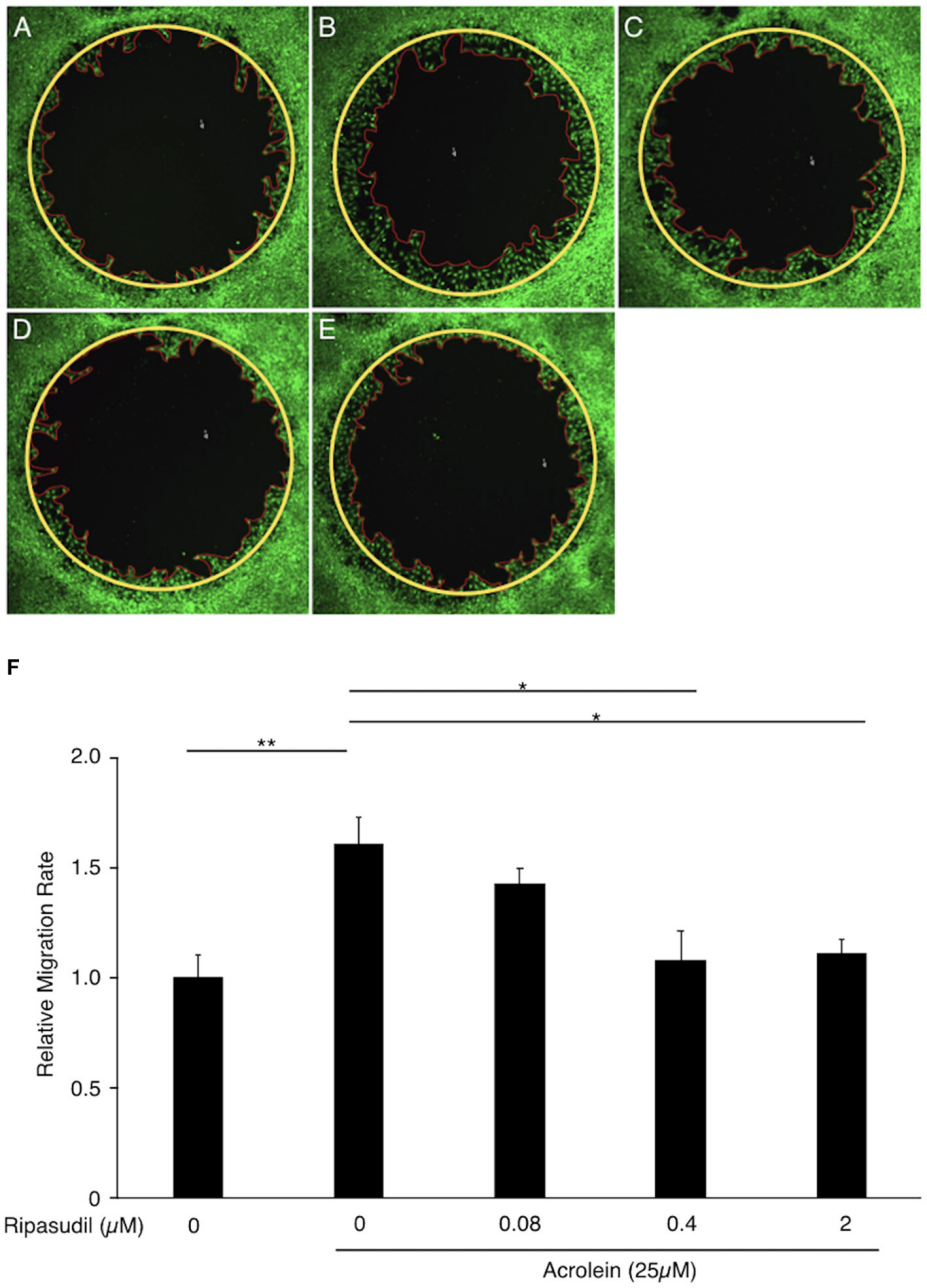
Impact of ripasudil administration on acrolein-induced migration of TR-MUL5 cells. Representative micrographs of TR-MUL5 cell migration after acrolein stimulation with or without ripasudil treatment. **(A)** Control. **(B)** Acrolein stimulation (25 μM). **(C)** Acrolein stimulation (25 μM) with ripasudil (0.08 μM). **(D)** Acrolein stimulation (25 μM) with ripasudil (0.4 μM). **(E)** Acrolein stimulation (25 μM) with ripasudil (2 μM). **(F)** Cell migration analysis in TR-MUL5 cells after acrolein stimulation with or without ripasudil (*n* = 4 each). Values represent the mean ± SEM; *P < 0.05, **P < 0.01.

To evaluate the association between ROCK1 and the migration of Müller glial cells, we performed a migration assay of TR-MUL5 cells after acrolein stimulation with or without ripasudil. Acrolein accelerated the migration of TR-MUL5 cells compared to the control (*n* = 4, *P* < 0.01), and ripasudil (0.4 μM, 2 μM) attenuated this increase in migration (*n* = 4, *P* < 0.05, Figure 7).

## Discussion

In the present study, we demonstrated that: (1) ROCK proteins are present in the cytosol of glial cells in the fibrovascular tissues of patients with PDR; (2) the unsaturated aldehyde acrolein increases the production of ROCK1, but not ROCK2, in cultured Müller glial cells; (3) acrolein enhances phosphorylation of MYPT1 and MLC2 through the ROCK1 cascade induced by oxidative stress; and (4) ripasudil, a specific inhibitor of ROCKs, hampers formation and migration of stress fibers in Müller glial cells. The current data provide new insights into the mechanisms of glial cell migration, a significant process in fibrovascular formation in eyes afflicted with PDR.

Glial cells have increasingly gained attention as one of the major participants in the progression of DR. While the activation of glial cells represents some of the earliest events in the pathogenesis of DR (16), macroglial cells are also implicated in promoting proliferative changes through VEGF secretion and internal limiting membrane degradation during the late stage of DR (17–19). Hence, elucidating the cellular behaviors of glial cells in advanced stages of DR contributes to further understanding of the pathological mechanisms underlying vision-threatening disease. We previously demonstrated that acrolein-conjugated proteins are localized in glial cells in the fibrovascular tissues of PDR patients (20). The current data further demonstrated that ROCK proteins are ubiquitously present in glial cells in fibrovascular tissues and that acrolein stimulation increased the production of ROCK1 in cultured Müller glial cells. Intriguingly, acrolein stimulation decreased the expression levels of ROCK2. In addition, the expression levels of ROCK-associated molecules including MYPT1 and MLC2 were not changed by acrolein stimulation. Whereas, our group previously showed that acrolein stimulation increases cell viability in cultured retinal glial cells (7), the current data clearly demonstrated that increase of ROCK1 protein is not simply attributed to the effect of acrolein to the cell viability in cultured retinal glial cells.

In ROCK proteins, ROCK2 is known as the predominant form of in ocular tissues (11). Since ROCK1 and ROCK2 exhibit a high degree of sequence homology, both kinases essentially share not only a wide variety of downstream substrates but also consequent cellular functions. In fact, the ROCK isoforms promote the rearrangement of actin-myosin cytoskeleton organization mediated by phosphorylation of downstream target proteins, including MYPT1 and MLC2 (21). However, using mouse embryonic fibroblast cells derived from ROCK1^−/−^ and ROCK2^−/−^ mice, it was elucidated that the roles of ROCK1 and ROCK2 are not strictly equal; i.e., ROCK1 is involved in destabilizing the actin cytoskeleton by regulating MLC2 phosphorylation and peripheral actomyosin contraction, whereas ROCK2 is required for stabilizing the actin cytoskeleton by regulating cofilin phosphorylation (21). Therefore, selective upregulation of ROCK1 by acrolein, which was demonstrated in the present study, indicates that acrolein facilitates a transition in the ROCK system to rearrange actin cytoskeleton and generate contractile force in Müller glial cells, eventually leading to glial cell migration.

Cigarette smoking is known to produce an estimated 10^17^ oxidant molecules per puff (22). Previous studies revealed that acrolein, a major cytotoxic factor in cigarette smoke extract, rapidly binds to GSH (23) and induces oxidative stress in the body (24–26). In line with these findings and our previous data (7), the current data also showed that acrolein reduces intracellular GSH levels in cultured Müller glial cells. In addition, acrolein-induced *Rock1* mRNA synthesis was remarkably suppressed by NAC, a thiol-containing antioxidant and precursor of GSH, in a dose-dependent manner. Furthermore, hydrogen peroxide, one of the representative reactive oxygen species, increased *de novo* synthesis of *Rock1*. The present data convincingly demonstrates that depletion of GSH by acrolein increases intracellular oxidative stress, thereby enhancing ROCK1 production in retinal glial cells.

Furthermore, although not quantified, acrolein appears to augment phosphorylation of MYPT1 and MLC2, both of which appears to be suppressed by ripasudil in cultured Müller glial cells. In line with the western blotting data, immunofluorescence microscopy also suggested that acrolein increased MLC2 phosphorylation and F-actin bundling, which are characteristics of stress fiber formation, in cultured Müller glial cells. Whereas, immunofluorescent staining of total MLC2 protein was not conducted in the present study, western blotting showed no significant change in the expression of total MLC2, indicating that the increased signal of p-MLC2 along with actin fibers in the TR-MUL5 cells exposed to acrolein, at least in part, due to the increase of MLC2 phosphorylation. In addition, since the possible increase in MLC2 phosphorylation and F-actin bundling was diminished by ripasudil, the present study demonstrated that ROCKs are the downstream effectors of acrolein.

Notably, the current study exhibited that glial cell migration induced by acrolein was suppressed by the non-selective ROCK inhibitor ripasudil. We previously reported that acrolein induces the production of the pro-inflammatory chemokine CXCL1 and promotes glial cell migration in an autocrine fashion (7). Enriching the previous findings, the present study revealed that ROCKs are involved in acrolein-induced glial cell migration. Furthermore, given the selective upregulation of ROCK1 by acrolein, ROCK1 might be the focus of future studies investigating the mechanism of glial cell migration regulated by acrolein.

There are several limitations to this study. First, there was a tendency toward decreasing migration rate in the acrolein-stimulated glial cells treated with 0.08 μM ripasudil administration, while identical concentration of ripasudil showed no inhibitory effect to the phosphorylation of MYPT1 and MLC2. Second, the decrease of *Rock2* in retinal glial cells stimulated with acrolein was intriguing; however, the physiological and/or pathological significance was not studied in the current study. Further investigation is warranted to obtain further mechanistic insights into non-muscle myosin phosphorylation regulation through ROCK1 signaling in glial cells.

In summary, our data shed light on the regulatory mechanism of Müller glial cell migration. The current results suggest that acrolein and its downstream molecule ROCK1 are attractive molecular targets for the prevention of fibrovascular tissue formation in PDR.

## Data Availability Statement

The raw data supporting the conclusions of this article will be made available by the authors, without undue reservation.

## Ethics Statement

The studies involving human participants were reviewed and approved by Institutional Review Committee of Hokkaido University Hospital. The patients/participants provided their written informed consent to participate in this study.

## Author Contributions

KN contributed to conception and design of the study. KF, MM, KK, and SY conducted the experiments. KF, MM, and KN wrote sections of the manuscript. SI revised the first draft of the manuscript. All authors contributed to manuscript revision, read, and approved the submitted version.

## Conflict of Interest

The authors declare that the research was conducted in the absence of any commercial or financial relationships that could be construed as a potential conflict of interest.

## Publisher's Note

All claims expressed in this article are solely those of the authors and do not necessarily represent those of their affiliated organizations, or those of the publisher, the editors and the reviewers. Any product that may be evaluated in this article, or claim that may be made by its manufacturer, is not guaranteed or endorsed by the publisher.
